# The effect of space arrangement between anterior teeth on their retraction with clear aligners in first premolar extraction treatment: a finite element study

**DOI:** 10.1186/s40510-023-00484-1

**Published:** 2023-09-25

**Authors:** Yuan Cao, Zhi-Wei Wang, Da Chen, Lu Liu, Deng-Xin Li, Ni Li, Si-Qi Ying, Xin Liu, Fang Jin

**Affiliations:** 1 State key Laboratory of Oral & Maxillofacial Reconstruction and Regeneration, National Clinical Research Center for Oral Diseases, Shaanxi Clinical Research Center for Oral Diseases, Department of Orthodontics, School of Stomatology, The Fourth Military Medical University, Xi’an, 710032 Shaanxi China; 2 Jiuquan Satellite Launch Centre, Jiuquan, 732750 Gansu China; 3 State key Laboratory of Oral & Maxillofacial Reconstruction and Regeneration, National Clinical Research Center for Oral Diseases, Shaanxi International Joint Research Center for Oral Diseases, Center for Tissue Engineering, School of Stomatology, The Fourth Military Medical University, Xi’an, 710032 Shaanxi China

**Keywords:** Clear aligner, Aligner deformation, Anterior teeth, Tooth extraction, Finite element study

## Abstract

**Introduction:**

Clear aligner therapy has become increasingly popular in recent years, although it has encountered several difficulties in premolar extraction treatment. These difficulties include anterior dentition, lingual tipping and extrusion. The design of the present clinical scheme usually set a tiny space between the anterior teeth before retraction in order to obtain an ideal outcome. The objective of our research was to analyze the effect of the existing spaces during retraction.

**Methods:**

Models including maxillary dentition without first premolars, maxilla, periodontal ligaments, gingiva, or aligners were constructed and imported to an ANSYS workbench. Five groups of models were created: without spaces and with 0.25, 0.50, 0.75 and 1.00 mm spaces between the anterior dentition. A 0.20 mm retraction step was applied to all the groups.

**Results:**

As the spaces between the anterior dentition increased, the bowing effect of the aligner caused by the passive forces decreased gradually. Accordingly, the degree of extrusion of the anterior dentition was alleviated significantly, while sagittal movement was reduced. However, the overall movement tended to be a bodily displacement rather than tipping. Meanwhile, maximum Von Mises stress of the periodontal ligaments (PDLs) was markedly decreased.

**Conclusion:**

These analyses indicate that spaces between the anterior dentition during anterior retraction are beneficial for decreasing the tendency for extrusion of the anterior dentition and require provision of anchorage. Appropriate spaces can be designed to lest the lingual tipping and extrusion effect of the anterior teeth while simultaneously reducing the maximum stresses on PDLs.

## Background

Clear aligner therapy (CAT) has become more common in recent years as a result of its notable advantages over fixed appliances, such as comfort and esthetics [1]. However, because of a lack of stiffness in clear aligner appliances, many difficulties can be encountered in premolar extraction cases when retracting the anterior teeth [2]. Research has shown that despite the worldwide popularity of CAT, the spread of its application in premolar extraction cases remains limited [3]. Retraction of the anterior teeth to improve a patient’s appearance is considered arduous in premolar extraction cases. The loss of incisor torque and extrusion of the anterior teeth [4], as well as mesial inclination of the posterior teeth [5], are relatively common problems.

Considerable research has been undertaken to solve these problems, including the use of altered geometries built in the aligner such as the “power ridge” [6, 7], updating of aligner materials to improve their properties [8], and a series of steps to cause tooth movement [9]. As a result, significant progress has been made in increasing the efficacy of CAT and reducing the drawback of aligners in premolar extraction cases. Of these techniques, we observed in computer-aided design applications, such as Aligner and iOrtho, that many cases of premolar extraction cases had tiny spaces between the anterior teeth before the retraction steps. Meng et al. showed that 0.5–1.0 mm spaces between the anterior teeth increased the wrap of the aligner [10], which helped increase the control of the aligners in extraction cases. Unfortunately, the detailed mechanism underlying the tiny spaces have not been fully elucidated.

Three-dimensional (3D) finite element analysis (FEA) is a computer simulation technique used widely in orthodontics to stimulate the stress and trends of teeth movement under different forces [11]. A recent study demonstrated that validated three-dimensional finite element models revealed many mechanisms of clear aligners [12]. The advantages of FEA are that it is timely, visualized, and repeatable [13] and as a result of a long period of orthodontic clinical research, FEA has recently become increasingly popular for optimizing treatment regimes.

In this study, we performed FEA to verify the effects of anterior dentition spaces on anterior teeth retraction. Using analysis of the biological mechanism of the aligners and dentition, we aimed to evaluate whether the spaces obtained among the anterior dentition were conducive to retraction of the anterior teeth in first premolar extraction cases. We also determined the best size of the space that would provide a biological basis for future clinical trials.

## Materials and methods

The base 3D FE model to simulate the extraction of the first premolar orthodontic treatment was obtained using cone beam computer tomography (CBCT). After importing the CBCT images of a healthy adult orthodontic patient with well-aligned dentition and a normal axial inclination of the incisors, a 3D base model of the alveolar bone and maxillary dentition was established using MIMICS 20.0 (Materialise, Leuven, Belgium) and GEOMAGIC Studio 2014 (3D Systems, NC, USA). The first premolars were removed to obtain an extraction dentition. The periodontal ligaments (PDLs) were modeled on root shapes using a thickness of 0.25 mm between the teeth and alveolar bone. As reported by a previous study, the aligners were modeled on crown shapes with a thickness of 0.5 mm [6, 11].

**Fig. 1:**
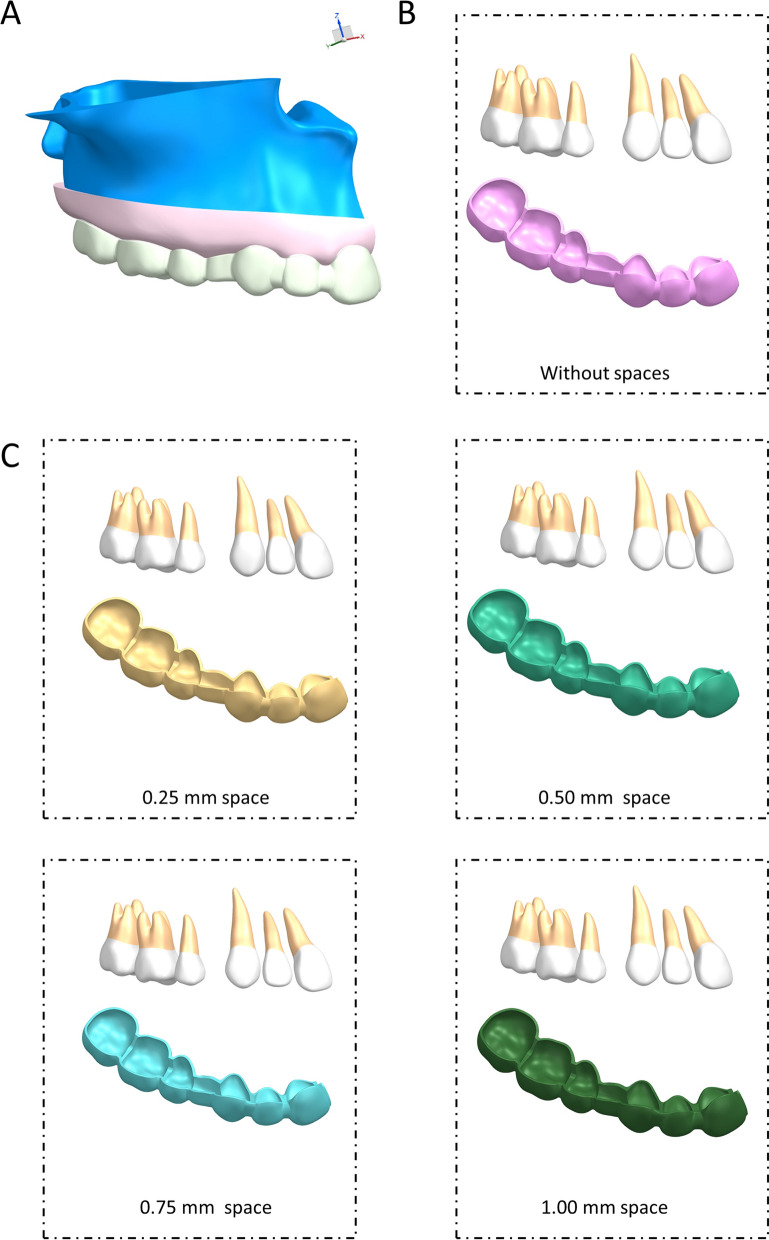
Computer-aided designed models. **A** The three-dimensional finite element model for anterior dentition retraction involved in the first premolar extraction. **B** The dentition arrangements model without spaces and the corresponding aligner model. **C** The dentition arrangements model with 0.25, 0.50, 0.75, and 1.00 mm spaces and the corresponding aligner model

The maxilla was then moved inward by 1.2 mm to generate a bone cortex and cancellous structure, or alternatively was moved outwards by 2 mm to generate a gingival structure. All the above models were established using NX1911 software (Siemens, **Nuremberg,** Germany). All the components were assembled and imported into the ANSYS Workbench 2019 (Ansys, Pennsylvania, USA) to generate a 3D FE model for FEA. The FE models are shown in Fig. 1A. All the components were set as linear elastic and homogeneous. No materials were used to fill the extraction space. As shown in Table 1, the mechanical properties were adapted from previous studies. The upper sections of the alveolar bone were set as the boundary regions, which meant that the maxilla was fixed when the force was loaded. Bonded contacts were set between the PDLs and teeth, the PDLs and alveolar bone, the gingiva and teeth, and the gingiva and alveolar bone. Surface-to-surface contact was used between the aligner surface and the teeth, with a friction coefficient μ = 0.1 (Fig. 1A).

**Table 1:** Properties of the materials considered in this study

Model	Young’s modulus (Mpa)	Poisson’s ratio
Cancellous bone	1370	0.3
Cortial bone	13,700	0.3
Teeth	19,613	0.15
Gingival	2.8	0.4
PDL	0.67	0.45
Clear aligner	528	0.36

Based on the above model (Fig. 1B), four protocols were set according to the space between the anterior teeth (0.25, 0.50, 0.75, and 1.00 mm; Fig. 1C). A retraction of 0.2 mm in the sagittal direction was applied to the anterior region to simulate a clinical event. The force was driven by the deformation of the aligner, with the results calculated by ANSYS Workbench 2019 software.

The direction of the dentition was described by the global coordinate system. The direction of the y-axis was the intersection of the sagittal plane and the occlusal plane, with the positive direction pointing posteriorly. The direction of the x-axis was coronally perpendicular to the y-axis, with the positive direction pointed to the left side of the patient. The direction of the z-axis was vertically perpendicular to the x-axis and y-axis, with the positive direction pointing apical. The mesial point, midpoint, and distal point of the incisal and the apical point of the root of the incisors were used as the measuring points. The midpoint of the mesial marginal ridge and the distal marginal ridge of the second premolars were used as the measuring points.

The sagittal displacement of the incisor’s crown was calculated as (sagittal mesial point displacement + sagittal midpoint displacement + sagittal distal point displacements), and the degree of tipping of the incisor was calculated as (sagittal displacement of the crown – sagittal displacement of the root)/ [(sagittal displacement of the crown + sagittal displacement of the root)/2]. The mesial inclination degree was calculated as (vertical distal marginal point displacement − vertical mesial marginal point displacement)/ sagittal displacement of the corresponding tooth).

## Results

### The vertical deformations of the clear aligner and dentition changed as the space changed

**Fig. 2:**
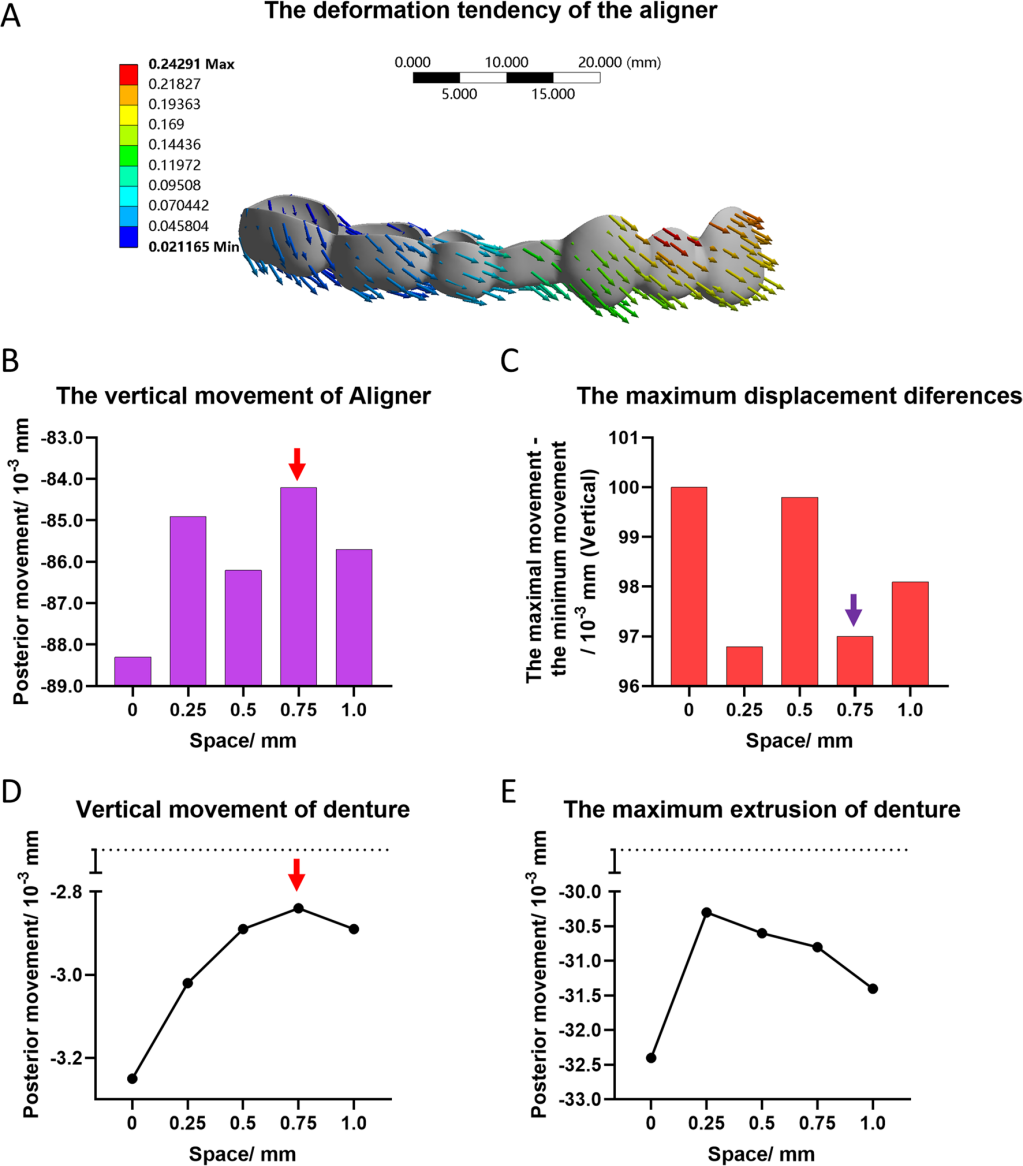
The vertical deformations of the clear aligners and the tendency for vertical displacement of the dentition in the groups with different spaces. **A** The deformation tendency of the aligner without spaces. **B** The values of minimum movement of the aligner in the vertical direction. **C** The values of maximum displacement differences of the aligner in the vertical direction. (The maximum displacement difference = maximum movement − minimum movement). **D** The average vertical movement of the whole dentition. **E** The tendency for maximum extrusion of the whole dentition in the vertical direction

As shown in Fig. 2A, when the anterior teeth retracted, displacements of the clear aligner in both the anterior and posterior extraction positions had a tendency to elongate. The extrusion tendency of the anterior segment was more obvious than that of the posterior segment. Beyond the pre-set coordinate system, the maximum vertical movements of the aligner away from the gingiva decreased as the space increased, with minimum vertical displacement observed in the 0.75 mm group (Fig. 2B). The maximum displacement discrepancy of the aligner is shown in Fig. 2C. The arrangements with spaces showed smaller discrepancies than those measured in the control. The groups with 0.25 mm and 0.75 mm spaces of the aligners also showed obvious minor deformation. The average vertical movements in the entire dentition showed less extrusion than the group without a space (Fig. 2D), with this effect tending to be minor in the 0.75 mm group, as shown by the red arrow. The maximum extrusion movements were also decreased in all the groups (Fig. 2E).

### Incisor retraction was associated with a concomitant decrease in the degree of extrusion of the anterior dentition in the groups with spaces

**Fig. 3:**
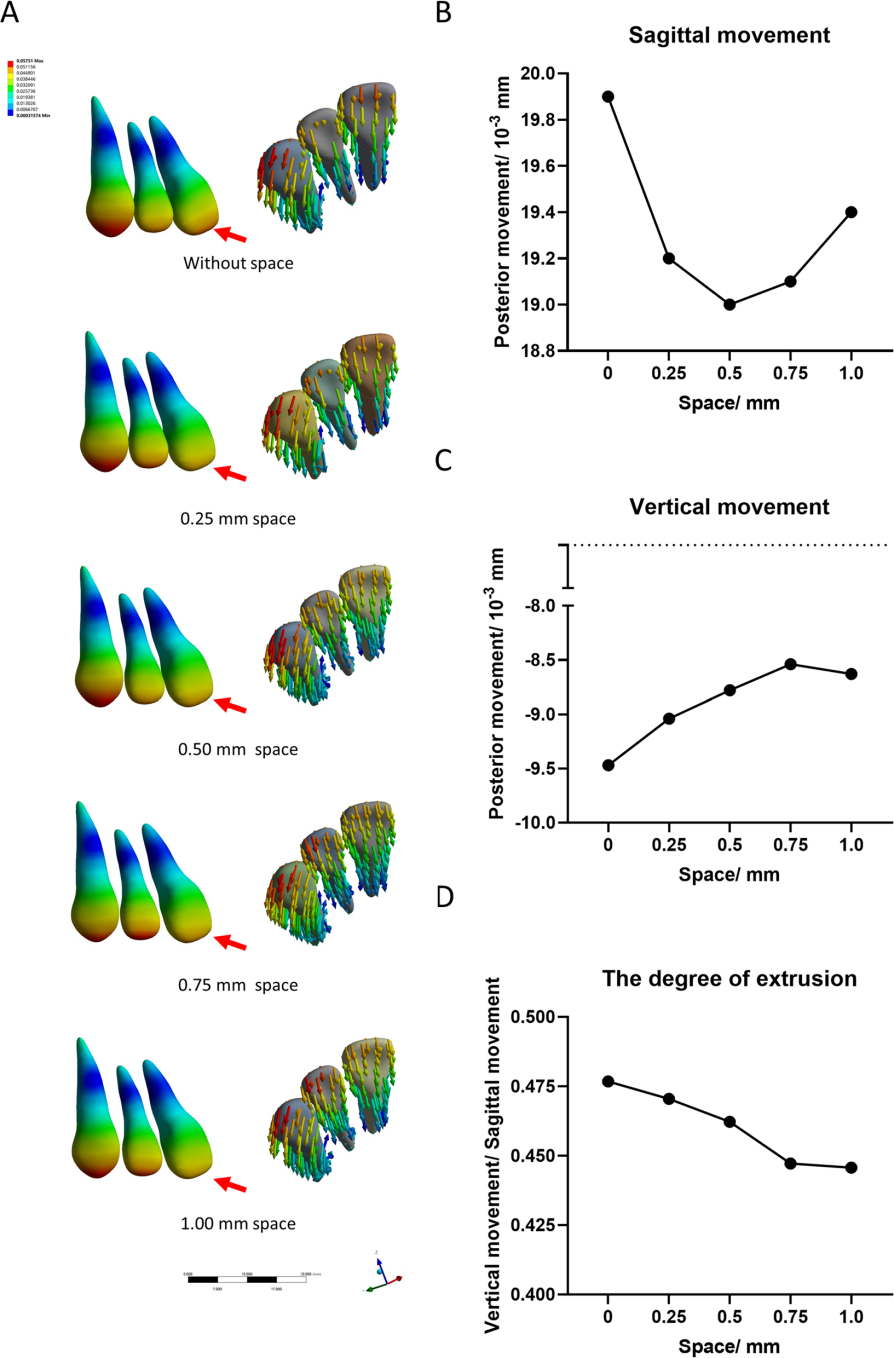
The tendency for displacement of the anterior dentition in the groups with different spaces. **A** The pattern of stress distribution of the anterior dentition and the direction of the tendency. **B** The anterior dentition’s tendency for displacement in the sagittal direction. **C** The anterior dentition’s displacement in the vertical direction. **D** The degree of extrusion under the conditions of similar sagittal movement. (The degree of the extrusion = vertical movement/sagittal movement)

Figure 3A shows the distribution of regional stress in the anterior dentition. Stress on the tip of the incisors and canine teeth decreased with the presence of spaces. The existence of spaces also resulted in the aligner showing a decreased tendency for sagittal movement (Fig. 3B), with the lowest sagittal movement observed in the model with a 0.5 mm space. When the space increased above 0.5 mm, the sagittal movement showed an increasing trend, but was still smaller than that in the control (i.e., without a space). In addition, an increase in the spaces resulted in a gradual decrease in vertical displacements manifested by extrusion of the anterior dentition (Fig. 3C). In general, as shown in Fig. 3D, under the same retraction distances, the tendency for anterior dentition extrusion was reduced with an increase in the size of the space, with this tendency being attenuated with a 0.75 mm space.

### The different spaces showed various trends of movement of individual incisors

**Fig. 4:**
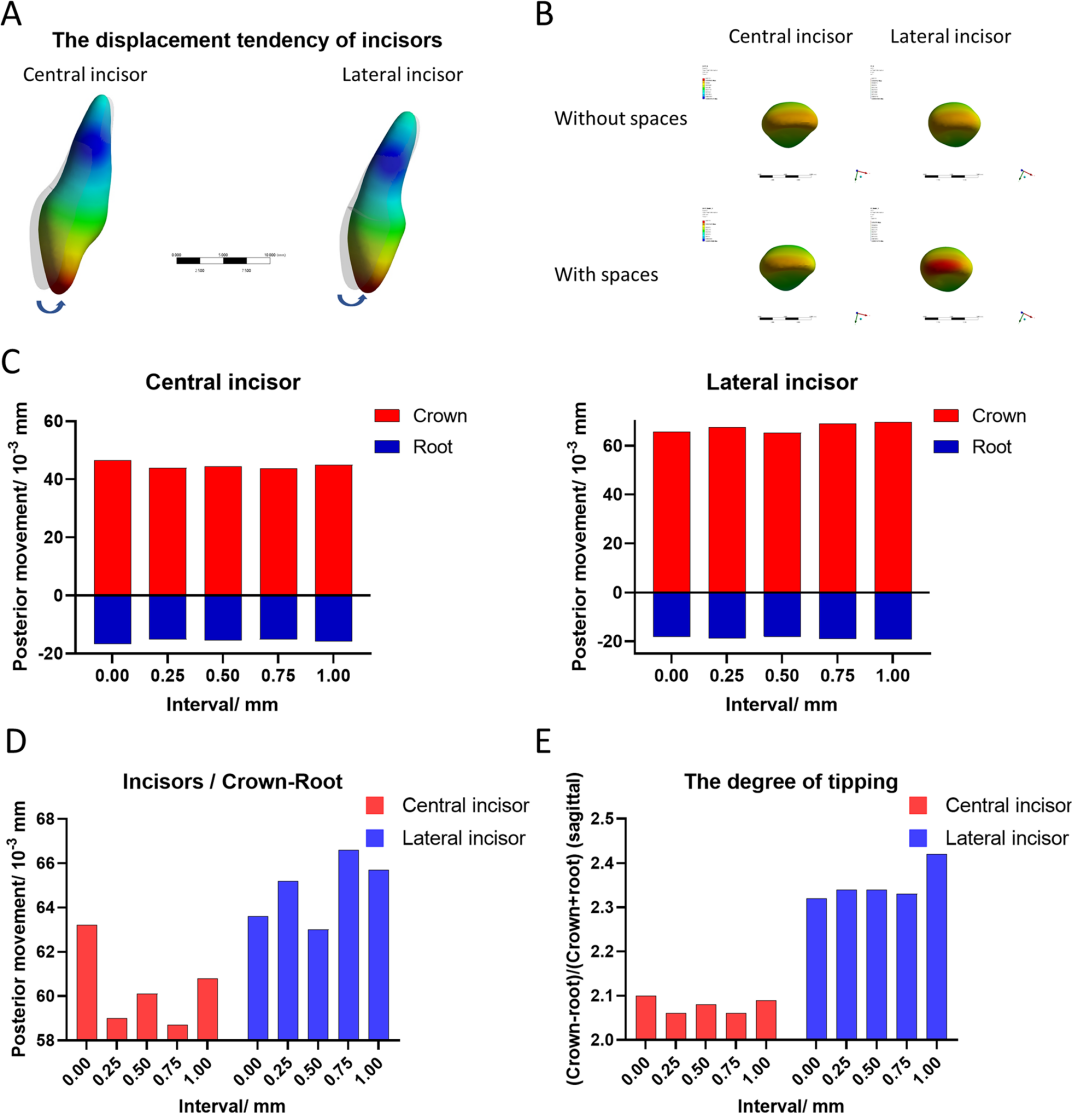
The stress distribution pattern of the individual incisors. **A** The arrows indicate the direction of movement of the central incisor (left) and lateral incisor (right). **B** The pattern of stress distribution of the coronal tips between groups with and without spaces. The red area represents the stress concentration sites. **C** Individual displacement of the crown and root of the central and lateral incisors. The red bar represents the crown, and the blue bar represents the root. **D** Comparison of tipping of the central and lateral incisors. The red column represents the central incisor, and the blue column represents the lateral incisor. **E** Comparison of tipping of the central and lateral incisors under the same sagittal displacement. The red column represents the central incisor, and the blue column represents the lateral incisor. The degree of tipping = (crown − root)/[(crown + root)/2]

**Fig. 5:**
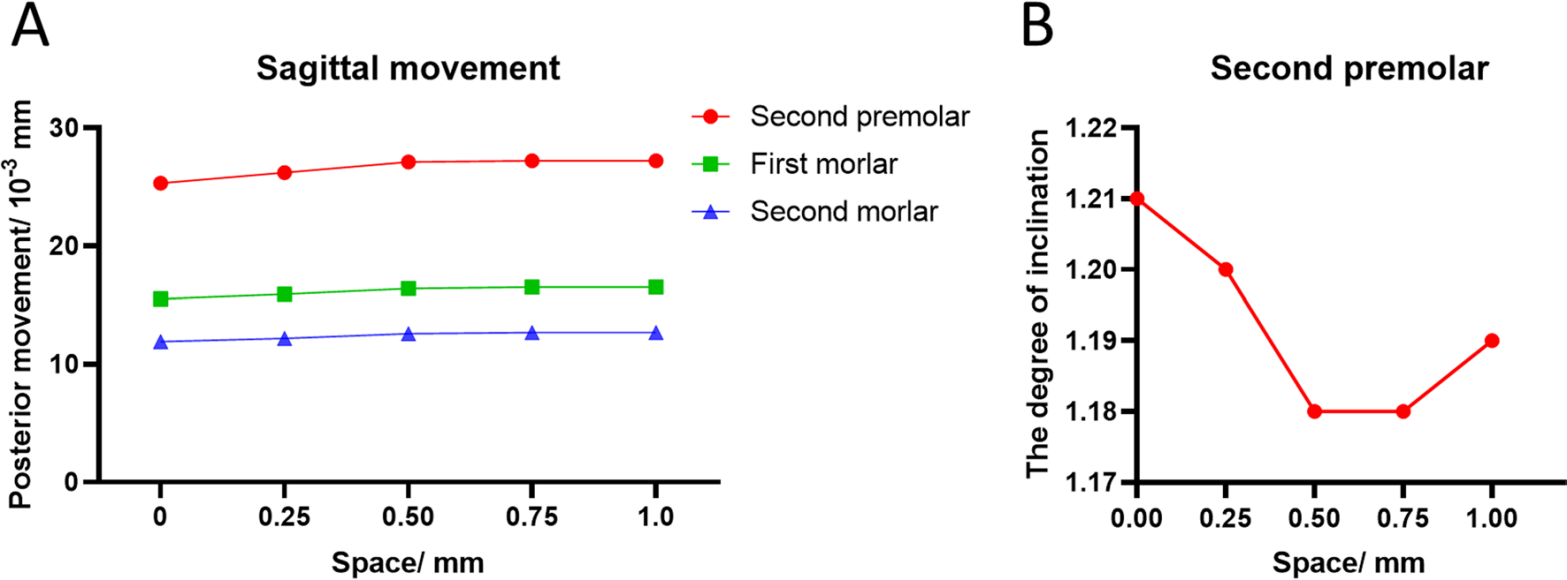
The tendency for individual anchorage teeth displacement. **A** The tendency for posterior movement of the second premolar, the first molar, and the second molar in the sagittal direction. The red line represents the second premolar, the green line the first molar, and the blue line the second molar. **B** The mesial inclination degree of the second premolar. The mesial inclination degree = (the vertical distal marginal point displacement − the vertical mesial marginal point displacement)/the sagittal displacement of the corresponding tooth

The arrow in Fig. 4A shows a trend of tipping of the individual anterior teeth under the experimental conditions. The incisal of the central incisor and canine showed less stress as the spaces increased. Meanwhile, more stress spread to the incisal of the lateral incisor (Fig. 4B). As shown in Fig. 4C, the displacement tendencies of the crown and the root in the sagittal direction of the incisors changed in the different groups. Tipping was represented by the difference between the displacement of the crown and the root. Fig. 5D shows the different values for displacement of the incisors that represented tipping. The red column represents the central incisor and the blue column represents the lateral incisor. The lateral incisors showed more obvious tipping than the central incisors. The minimum tipping of the central incisors was observed in the 0.75 mm group, while minimum tipping of the lateral incisors occurred in the 0.5 mm group. Figure 5E shows the degree of tipping for the different sagittal movements of the teeth and the difference between displacement of the crown and root under the same mean sagittal movement of the teeth. The minimum degree of tipping of the central incisor occurred in the 0.75 mm group, while the minimum degree of tipping of the lateral incisor was also observed in the 0.75 mm group.

### The different spaces showed various trends of movement of the anchorage teeth

The differences in anchorage between the experimental groups are shown in Fig. 5A. The loss of all the anchor teeth increased subtly. However, the mesial inclinations of the second premolar were alleviated under the same sagittal displacement (Fig. 5B), with the smallest mesial inclination observed in the 0.75 mm group.

### The position of the highest Von Mises stress of PDLs changed with alterations in the spaces, the maximum Von Mises stress of the PDLs, and the average Von Mises stress of the alveolar bone

**Fig. 6:**
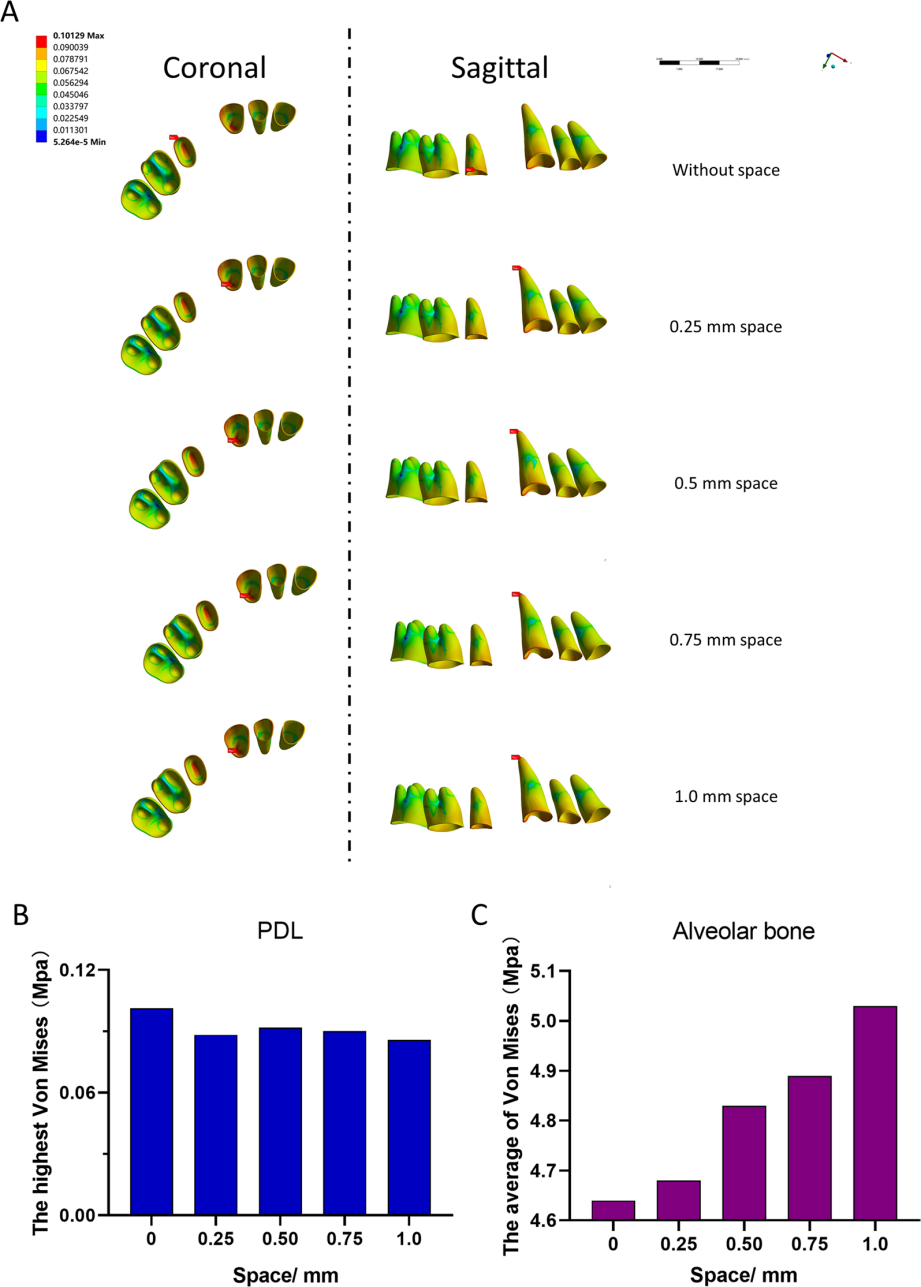
The distribution of Von Mises stress in the different experimental models. **A** The distribution of Von Mises stress of the PDLs in the whole dentition, with the position of maximum Von Mises stress shown with a red label. **B** The maximum values of Von Mises stress of the PDLs. **C** The average values of Von Mises stress of the alveolar bone

Figure 6A shows the pattern of regional stress distribution of the whole dentition’s PDLs in the sagittal and coronal planes. The highest Von Mises stress of the PDL occurred between the cervical of the second premolar to the apical of the canine. The magnitude of this stress also decreased significantly compared with that measured in the control, with the lowest stress observed in the group with a 1.0 mm space (Fig. 6B). In contrast, as the distance of the space increased, the average von Mises stress of the alveolar bone showed a rising gradient (Fig. 6C).

## Discussion

CAT has gained considerable popularity because of its comfort and aesthetics compared with those obtained using traditional fixed appliances [1]. However, the application of CAT to extraction of first premolar cases is still met with many difficulties. Lingual tipping and extrusion of the incisors, named “the pendulum effect,” combined with mesial inclination of the posterior dentition, named “the roller-coaster effect”, are the most troublesome obstacles to completing an extraction of the premolars case [14]. Researchers consider the reasons for these phenomena are that application of the aligner to the crown of the teeth and lack of root control may cause a tipping movement of the anterior teeth during application of retracting forces [15]. As shown in Fig. 3, this possibility was consistent with our results. We showed that when solo retraction forces were applied, the anterior dentitions showed sagittal retraction combined with extrusion, that is, lingual tipping of the incisors (Fig. 4). Subsequently, the aligners were subjected to passive forces (Fig. 2A). Due to Newton's third law of motion, the whole aligner tended to extrude, with the front of the aligner separated by the extraction areas being more obvious than the rear. Unfortunately, due to the lack of stiffness of the aligner and interruption of the extraction sites, it was easy to produce a bending effect according to the displacement difference of the appliances, thereby theoretically causing deformation. The shortened sagittal length of the aligner may then exacerbate these trends in teeth movement. Based on these findings, reducing the passive deformation of the aligner, or strengthening the strength of the aligners would solve this problem. A study by Danielle [5] showed no significant difference in the degree of dental tipping around the extraction space between soft and hard aligners. In the present study, we showed that the appearance of spaces between the anterior dentition may reduce passive aligner deformation. Figure 2B shows the distance of the maximal extrusion sites of the aligners. Meanwhile, Fig. 2C shows the differences between the maximum and minimum displacement of the aligner, indicating a trend toward vertical deformation. Due to the aligner being an inseparable whole, smaller differences resulted in lower bending effects. Conjecture regarding this trend is that as the space increases, the position of the space can become a fulcrum, thereby reducing deformation of the aligner. However, a space that is too large would counteract the above effect. In this regard, our research also demonstrated that the average vertical movement of the whole dentition was extrusion, with the group with a 0.75 mm space showing a minimal degree of extrusion (Fig. 3D). Furthermore, the maximum vertical movement of the whole dentition in extrusion was smallest in the 0.25 mm group (Fig. 3E). Taken together, these results demonstrate that a proper space between the anterior dentition has advantages in retraction by reducing unexpected movements of the teeth.

Recently, the majority of methods to resolve pendulum and roller-coaster effects involve either the use of a power ridge [6], overtreatment of the aligners [11], or an intrusion along with retraction [15]. Most of this research attempted to acquire a tendency for bodily movement. However, according to the above studies [6, 10, 14], even with the use of new materials or attachments, it is somewhat difficult to achieve the desired root control [16]. As shown in Fig. 4A, our research acquired the same consequence, namely a trend for incisor movement with inclination rather than bodily movement (Fig. 4C). A study by Danielle [5] reported greater tipping around the extraction of the second premolar than around the first premolar, while Crossman and Reed [17] showed that second premolar extraction sites in the maxilla had more unsatisfactory contacts than first premolar extraction sites. These studies indicated that the arrangement of the dentition around the extraction may change the biochemical torque of the aligner. In clinical practice, professional doctors tend to use two-step movement (“frog-jump” models) instead of en masse movement to carry out tooth retraction. This mimics the fixed appliance and is conducive to anchorage protection and control of the anterior teeth. Hennessy et al. compared the inclination of the mandibular incisor between the aligner and fixed appliances and found no difference in controlling the incisor’s torque during non-extraction treatment [18]. However, in extraction treatment, the aligners showed a major lack of anterior dentition control compared with that achieved using fixed appliances [19]. Hennessy et al. [17] hypothesized that conduction of the aligner forces may be interrupted by the extraction sites. In our experimental models (Fig. 1C), the spaces between the anterior dentition decreased the space between the posterior and the anterior dentition. The trends of anterior dentition movement shown in Fig. 3 indicate less lingual inclination. Even though the trends of the sagittal movement decreased significantly compared with those of the model without a space, the relative extrusion of the anterior dentition decreased significantly under the conditions of the same sagittal movement. This decreasing trend flattened in the group with a 0.75 mm space. Meanwhile, the stress on the central incisor and canine was well distributed, although the tip of the lateral incisor bore more stress (Fig. 4B). Compared with the distribution among incisors and canines, greater stress on the tip of lateral incisors resulted in greater settings for correction, such as adding more root torque. This occurred more available because the position of the central incisor and canine restricted the movement of the lateral incisors, with the whole length of the aligner being consistent. Based on the above experimental results and conjecture, we consider that the arrangement of dentition may alter biochemical forces in the system. Therefore, as shown in Fig. 4E, the degree of tipping under the same sagittal retraction was greatly improved in both incisors.

According to our results, the presence of spaces appears to require more anchorage to retract the anterior teeth. As shown in Fig. 5A, the trends of sagittal movement of the posterior were increased, whereas the degree of inclination of the second premolar mesial was decreased (Fig. 5B). This trend was the greatest in the group with a 0.75 mm space. In our study, the increased sagittal movement meant that the experimental groups needed more anchorage, indicating that the displacement tendencies of the anterior teeth were a bodily movement, due to this requiring more elasticity force than that required for inclination [20]. Auxiliary anchorage such as the use of a Miniscrew or intermaxillary traction was applied routinely in the treatment of the extracted teeth to protect the posterior teeth from mesial movements. No additional anchorage was required with the presence of spaces among the anterior teeth if regular anchorages were provided. Meanwhile, because the central incisor showed only a small tendency for sagittal displacement, we provided more forces at every step of the aligner.

Root resorption often occurs in clinical orthodontic treatment [21]. Although the incidence of root resorption in CAT is lower than that for fixed appliances [22], there is still a risk of this occurring. Research has shown that hydrostatic pressure exceeds typical human capillary blood pressure in the PDLs, resulting in an increased risk of root resorption [23]. Therefore, we paid attention to the Von Mises stress of the PDLs. The results of PDL stress in the FEA as well as the data of our research showed a greater spread regardless of the clear aligner or fixed appliances [6, 11, 15, 24]. Maxillary central incisors were most susceptible to root resorption. As shown in Fig. 5, there was no additional risk in this procedure. However, more biological research needs to be carried out in the future to confirm these possibilities.

The main aim of our experiments was to examine the consequence of the tendency for tooth displacement in the spaces between the anterior dentition during application of the retraction forces. Analysis of the FEA results will assist in preparing an optimal protocol for further clinical trials and clinical practice. We recommended that proper spaces (0.75 mm) should be used to avoid some side effects of clear aligner deformation in premolar extraction treatments. It should be emphasized that more research, such as clinical trials and mechanical trials needs to be conducted to confirm our conclusion.

## Limitations

FEA lacks the consideration of biological components as an engineering method analysis. Each orthodontic case had different conditions, such as the inclination of the teeth, periodontal conditions, and oral environment. This is more complicated in clinical conditions. Therefore, more clinical studies are needed to support our results. On the other hand, the FEA is good at analyzing the transient force and movement of the teeth. However, the intricacy of the activations of tooth movement and retention involved many factors. Whether the transient movement reflected the real condition needs more experimental confirmation.

## Conclusions

1. The spaces between the anterior teeth altered the shape of the aligner, thereby resulting in a lower dentition extrusion effect.
2. Appropriate spaces between the anterior teeth (e.g., 0.75 mm in this model) were beneficial for preventing tipping of the incisors.
3. The use of spaces to retract the anterior teeth needs to pay attention to anchorage.

## Data Availability

The datasets used and/or analyzed during the current study are available from the corresponding author on reasonable request.

## Abbreviations

CAT: Clear aligner therapy
3D: Three-dimensional
FEA: Finite element analysis
FE: Finite element
PDLs: Periodontal ligaments
CBCT: Cone beam computer tomography
